# Discovery, Antitumor Activity, and Fermentation Optimization of Roquefortines from *Penicillium* sp. OUCMDZ-1435

**DOI:** 10.3390/molecules28073180

**Published:** 2023-04-03

**Authors:** Xingxing He, Yanzheng Jin, Fei Kong, Liyuan Yang, Mingzhuang Zhu, Yi Wang

**Affiliations:** 1 School of Medicine and Pharmacy, Ocean University of China, Laboratory for Marine Drugs and Bioproducts of Qingdao National Laboratory for Marine Science and Technology, Qingdao 266003, China; 2 Fisheris College, Ocean University of China, Qingdao 266003, China

**Keywords:** meleagrin, oxaline, anticancer, fermentation, *Penicillium*

## Abstract

Meleagrin and oxaline, which belong to the roquefortine alkaloids with a unique dihydroindole spiroamide framework, have significant bioactivities, especially tumor cell inhibitory activity. In order to discover the requefortine alkaloids, *Penicillium* sp. OUCMDZ-1435 was fished and identified from marine fungi using molecular probe technology. Meleagrin (**1**) and oxaline (**2**) were isolated from it. In addition, we first reported that compounds **1** and **2** could effectively inhibit the proliferation and metastasis of the human HepG2 cell and induce HepG2 cell apoptosis and cell cycle arrest in the G2/M phase. Additionally, the fermentation of Meleagrin (**1**) was optimized to increase its yield to 335 mg/L. These results provided bioactive inspiration and fungus resources for roquefortine alkaloid development.

## 1. Introduction

Roquefortine alkaloids are a class of metabolites produced mainly by *Penicillium* fungi. The structure of these compounds is composed of histidine, tryptophan, and isovaleric acid residues [1]. These alkaloid families included four major groups: roquefortines [2,3,4,5,6,7,8,9], meleagrins [3,9,10], glandicolines [11], and oxalines [12,13]. They possessed complex structures and impressive bioactivities; the meleagrin family was reported to have cytotoxicity against HL-60, A-549, BEL-7402, and MOLT-4 cell lines and inhibited tubulin polymerization to cause cell cycle arrest in the G2/M phase when applied to A-549 and HL-60 [3,9]. Oxaline can inhibit tubulin polymerization in Jurkat cells, resulting in cell cycle arrest at the M phase [14]. Roquefortine C is the key biosynthetic precursor in over 30 different Penicillium species, and the biosynthesis processes involving it have been clarified to involve a dimodular nonribosomal peptide synthetase and other associated genes. The downstream metabolites, glandicolines, meleagrin, oxaline, and neoxaline, derived from roquefortine C, which were not found in all reported roquefortine-like alkaloids-producing fungi, have a unique triazaspirocyclic skeleton [15,16,17]. Post-modification mainly occurred on the -NH of the indole ring and imidazole ring, such as meleagrin and oxaline [7,18]. The meleagrins have attracted extensive attention for their significant bioactivities.

The Japanese Toshiaki Sunazuka research group carried out the total synthesis of indole alkaloids containing a unique dihydroindole spiroamide skeleton, including neox-aline, oxaline, meleagrin, and their analogues, through the stereoselective introduction of the reverse isoprene group, benzyl carbon, and dihydroindole, to indole spirocyclic amine, and finally through more than 60 steps [13]. The synthesis process is extremely difficult, so investigating biological resource strains for biological fermentation shows great significance. As a part of natural resources, marine fungi have become an important resource for the development of antitumor drugs due to their novel structure and unique activity. Herein, three pairs of primers were designed as probes to search for marine-derived fungi. Therefore, we discovered *Penicillium* sp. OUCMDZ-1435 was a target strain. Additionally, we first reported that compounds **1** and **2** could effectively inhibit the proliferation and metastasis of human HepG2 cells and induce HepG2 cell apoptosis and cell cycle arrest in the G2/M phase. In order to improve the yield, we optimized the fermentation of 1 and increased its yield to 335 mg/L.

## 2. Results

### 2.1. The Target Fungal Strain

**Figure 1:**
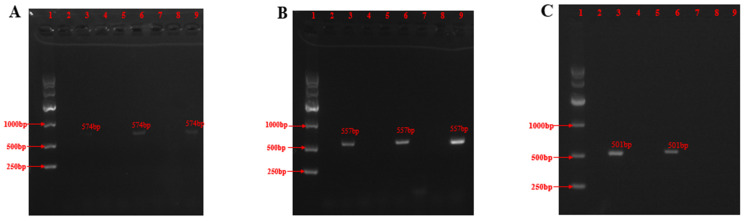
PCR amplification of genes required for biosynthesis in different strains. (**A**) PCR amplification of the RDS gene in different strains. Lane 1, Marker; Lane 2, Water; Lane 3, OUCMDZ-1435; Lane 4, OUCMDZ-3597; Lane 5, OUCMDZ-4032; Lane 6, OUCMDZ-4014; Lane 7, OUCMDZ-5210; Lane 8, OUCMDZ-019; and Lane 9, OUCMDZ-4754. (**B**) PCR amplification of the RPT gene in different strains. (**C**) PCR amplification of the SRO gene in different strains.

Based on the analysis of the biosynthetic pathway of roquefortines alkaloids (Figure S2), RDS-F/D primers (Table 1) were used to amplify histidine- and tryptophan-linked dipeptide synthases. RPT-F/R primers (Table 1) were used to amplify acetyltransferases required for the conversion of Cyclo-Trp-dehydroHis to Roquefortine C [19]. SRO-F/R primers (Table 1) were used to amplify the oxidase conversion of Roquefortines to Meleagrin/Oxaline. As a result, three fungal strains were fished for roquefortine alkaloids successfully. The OUCMDZ-1435 strain was selected as the target strain for the following experiment (Figure 1).

### 2.2. Isolation and Identification

The extract of the mycelium of *Penicillium* sp. OUCMDZ-1435 fractionated by VLC, Sephadex LH-20, and preparative HPLC columns provided compounds **1** and **2**.

**Figure 2:**
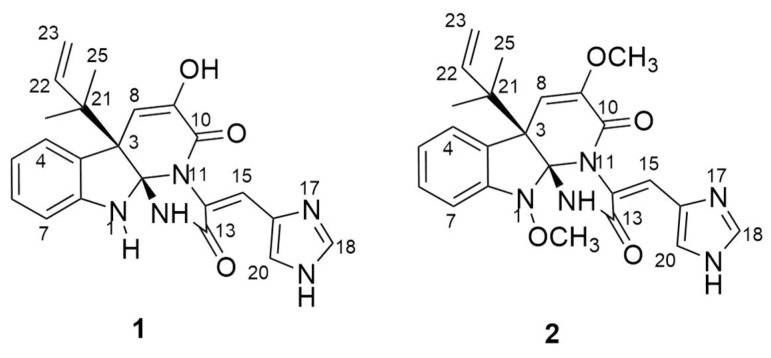
Chemical structure of compounds **1** and **2**.

Compound (**1**) was composed of a pale-yellow amorphous powder. The molecular formula was determined to be C_23_H_23_N_5_O_4_ by the ESI-MS analysis (*m*/*z* 434.2[M + H]^+^). Comparing the NMR data (Figures S3 and S4, Table S1 in Supplementary Material) with the literature, it was determined that the planar structure of compound **1** was consistent with the literature. Its UV spectrum displayed characteristic peaks at [λmax (logε): 199.2 (2.40), 229.8 (1.50), 285.4 (0.55), and 346.3 (1.50) nm]. The optical rotation value of compound **1** was [α]^25^D -61.2 (c 0.1 mg/mL, MeOH), and the literature review showed that the compound was consistent with the meleagrin reported in the literature. Thus, compound **1** was determined to be meleagrin (Figure 2).

Compound (**2**) was a pale-yellow amorphous powder with a molecular formula of C_24_H_25_N_5_O_4_ as deduced from the ESI-MS analysis (*m*/*z* 448.2 [M + H]^+^). Comparing the NMR data (Figures S5 and S6, Table S1) with the literature, it was determined that the planar structure of compound 2 was consistent with the literature. Its UV spectrum displayed characteristic peaks at [λmax (logε): 199.2 (2.40), 229.8 (1.50), 285.4 (0.55), and 346.3 (1.50) nm]. The optical rotation value of compound 2 was [α]^25^D -41.2 (c 0.1 mg/mL, MeOH), and the literature review showed that the compound was consistent with the oxaline reported in the literature. Thus, compound **2** was determined to be oxaline (Figure 2).

### 2.3. Biological Activity

#### 2.3.1. Cytotoxicity

In Table 2, meleagrin (**1**) and oxaline (**2**) were evaluated for their cytotoxicity against A549, K562, MCF-7, HepG2, P6C, and HCT-116 tumor cell lines and the human embryonic liver cell line L-02, with adriamycin as a positive control. The meleagrin showed inhibitions on the MCF-7, HCT-116, and HepG2 cells with IC_50_ values of 4.94, 5.7, and 1.82 μM. Notably, melegarin and oxaline displayed excellent cytotoxic activity against HepG2 cells with an IC_50_ value of 1.82 and 4.27 μM, respectively. The mechanism of cytotoxicity against the HepG2 cells deserves further research. The detailed results have not been reported.

#### 2.3.2. Cell Proliferation and Migration Assay

**Figure 3:**
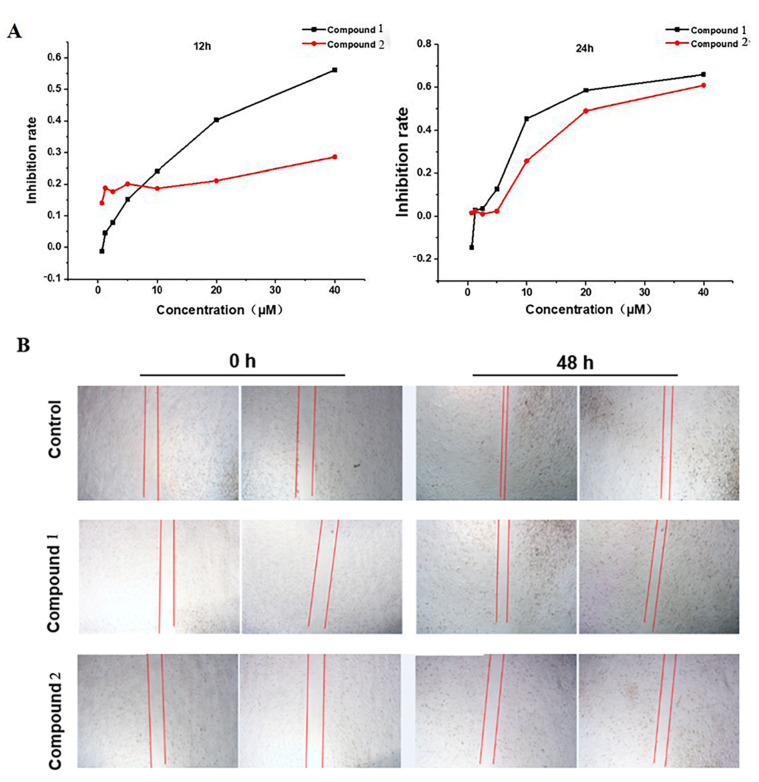
Cell proliferation and migration assay. (**A**) The inhibition rate of cell proliferation in HepG2 cells transfected with **1** and **2** was measured by MTT assay at 12 and 24 h. (**B**) A cell scratch assay evaluated the cell migration activity of compounds **1** and **2** by wound healing after 48 h.

Meleagrin (**1**) and oxaline (**2**) exhibited excellent cytotoxic activity against HepG2 cells; in particular, their ability to inhibit tumor cell proliferation and migration was further evaluated [20,21,22]. The compounds at seven different concentrations (0.675, 1.25, 2.5, 5, 10, 20, and 40 μM) were treated with HepG2 cells for 12 h and 24 h; the MTT results are shown in Figure 3A, and two compounds significantly inhibit the proliferation of HepG2 cells with a certain concentration dependency. The half-inhibited concentrations of **1** and **2** on HepG2 cells for 24 h were 18 μM and 25 μM, separately. The effects of the two alkaloid compounds on cell migration in HepG2 cells were evaluated by the wound scratch assay; the results of the treatment at 0 and 48 h were as follows (Figure 3B): the wound widths of the cells treated with **1** and **2** were wider than the control group cells after 48 h, two compounds significantly prevented cell migration. At the same concentration of 1 μM, compared with meleagrin (**1**), oxaline (**2**) had a stronger inhibition effect on the wound healing of liver cancer cell scratches. The above results demonstrated that meleagrin and oxaline can effectively block the proliferation and migration of HepG2 cells.

#### 2.3.3. Cell Cycle Arrest Assay

**Figure 4:**
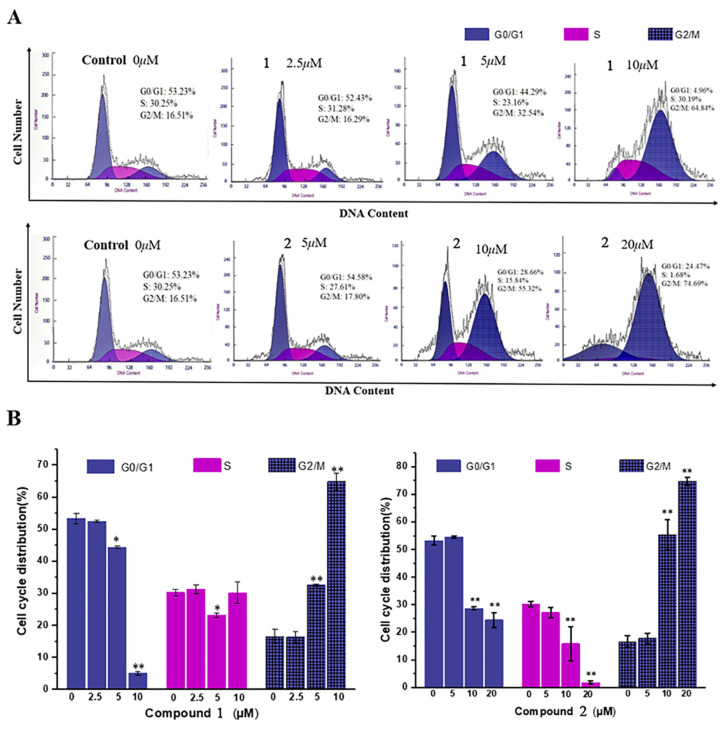
Effect of compounds **1** and **2** on the cycle of HepG2 cells. (**A**) HepG2 cells were treated with compounds **1** (0, 2.5, 5, and 10 μM) and **2** (0, 5, 10, and 20 μM) for 24 h, and then the cells were fixed, stained with PI, and analyzed by flow cytometry. (**B**) The quantification of cell cycle distribution. Data are the means ± SD of three independent experiments. * *p* < 0.05, ** *p* < 0.01, vs. control group.

The cell cycle is usually highly conserved and possesses the critical function of regulating the biological system that controls cellular proliferation and differentiation. Cancer is a characteristic of an uncontrolled cell cycle along with the unregulated proliferation of tumor cells; hence, cell cycle arrest is a promising strategy used to stop cancer cell proliferation [23]. Compounds **1** and **2** experienced good growth inhibition toward the HepG2 cell line and were chosen to assay the effect on the cell cycle. Compound **1** at concentrations of 0, 2.5, 5, and 10 μM and 14 at concentrations of 0, 5, 10, and 20 μM were separately treated with HepG2 cells for 24 h, and then the cells were harvested, washed, fixed, and stained with propidium iodide (PI). DNA was observed using flow cytometry for cell cycle DNA content analysis to calculate the distribution of the cell cycle in the G0/G1, S, and G2/M phases. As shown in Figure 4A, compared with the control group, compounds **1** and **2** were able to arrest HepG2 cells in the G2/M phase and exhibit a certain concentration dependency. Along with the increase in the treated concentration, the proportion of cells in the G0/G1 phase was obviously reduced, while the percentage of cells in the G2/M phase was significantly increased, and the difference was statistically significant (*p* < 0.05). Compared with a 16.5% proportion of the G2/M phase in non-treated control cells, the G2/M phase cell percentage increased to 64.84% and 74.69% in cells treated with **1** at 10 μM and **2** at 20 μM (Figure 4B).

#### 2.3.4. Cell Apoptosis Assay

**Figure 5:**
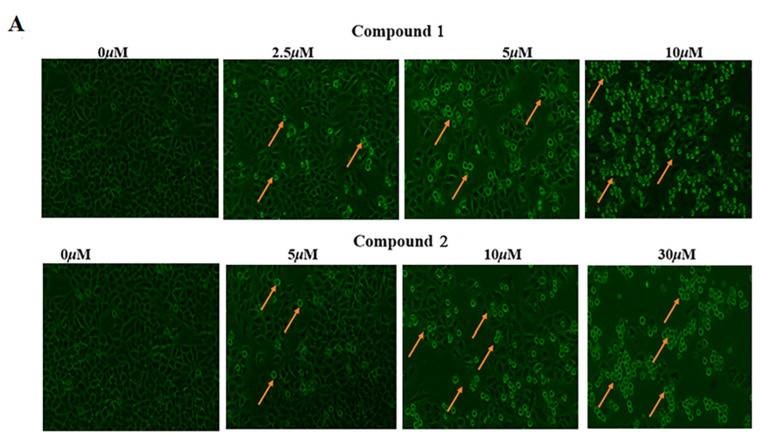

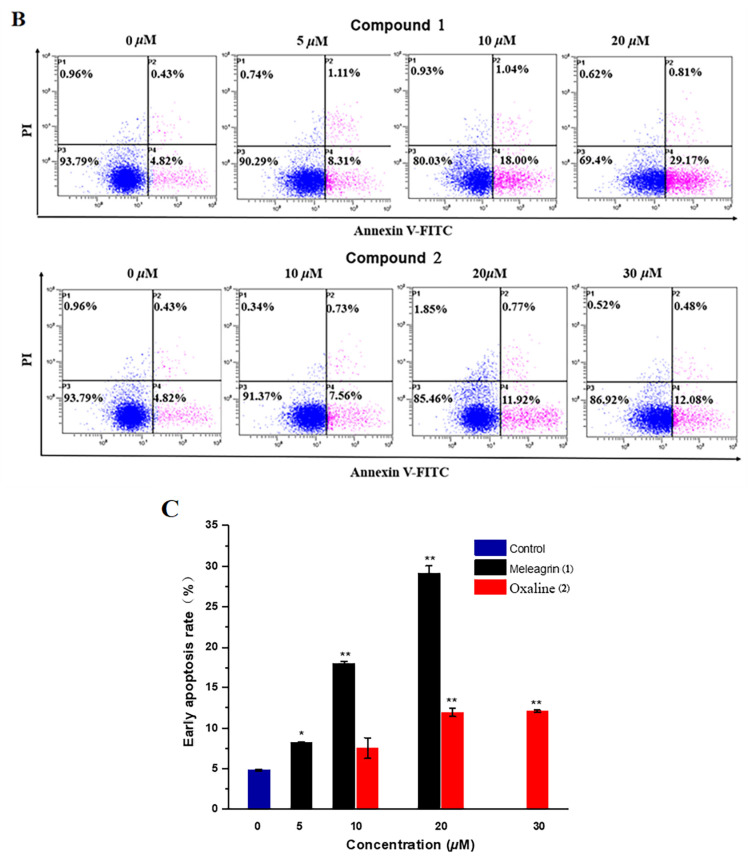
Meleagrin (**1**) and oxaline (**2**) induced an apoptosis rate in HepG2 cells. (**A**) Morphological changes in HepG2 cells were observed with microscopy (400× magnification). (**B**) On the flow cytometry is a two-parameter scatter plot: the lower left quadrant shows live cells (LL: Annexin V-/PI-); the lower right quadrant is the early apoptotic cell (LR: Annexin V /PI-); the upper right quadrant is the late apoptotic cell (UR: Annexin V /PI); and the upper left quadrant is a non-live cell and necrotic cell (UL: Annexin V-/PI). (**C**) The quantification of early cell apoptosis. Data are the means ± SD of three independent experiments. * *p* < 0.05, ** *p* < 0.01 vs. control group.

Apoptosis, as a process of procedural cell death, can eradicate damaged cells and prevent tumor growth to maintain the integrity of normal tissues and organs in response to DNA damage, cellular stress, or oncogene expression, and apoptosis can act as a vital target for the elimination of cancer cells [24,25]. As Figure 5A exhibited, compared with the non-treated group, compounds **1** (2.5, 5, and 10 μM) and 2 (5, 10, and 20 μM) were separately treated with HepG2 cells, which all induced an obvious cell morphology change, such as cell shrinkage and rounding and membrane blebbing, which proved that compound **1** and **2** could induce apoptosis in HepG2 cells in a dose-dependent manner. The ability of compounds to induce apoptosis was further researched, and the apoptosis of HepG2 cells treated with different concentrations of compounds for 24 h was detected through a biparametric cytofluorimetric analysis using flow cytometry and an Annexin V-FITC/PI double-staining cell apoptosis detection kit. As can be seen in Figure 5B, along with the increase in concentration of compounds treated with human HepG2 cells, the apoptotic rate increased in a dose-dependent manner, especially the early apoptotic rate. Compared with the control group, which expressed an early apoptotic rate of 4.82 (Figure 5C), the apoptotic rates of HepG2 cells treated with compound **1** at 20 μM and compound **2** at 30 μM were 29.17% and 12.08%, respectively.

According to the results of the bioactivity evaluation, meleagrin (**1**) showed a wide range of cell proliferation inhibition, and oxaline (**2**) exhibited good cytotoxicity that was selective against the HepG2 cell. It can be concluded that meleagrin and oxaline can effectively inhibit the proliferation and migration of human HepG2 cells, and oxaline significantly inhibits the healing of cancer cell HepG2 scratches at a concentration of 1μM. Moreover, two compounds were able to induce G2/M phase cell cycle arrest and the apoptosis of HepG2 cells in a certain concentration-dependent manner. In view of the good biological activity of compound **1**, we decided to increase its yield through fermentation optimization.

### 2.4. Meleagrin Standard Curve

As mentioned above, the chemical synthesis of these compounds is extremely difficult, and the above tumor cell inhibitory activity also aroused our great interest. Therefore, we investigated the fermentation optimization of the main metabolite meleagrin.

**Figure 6:**
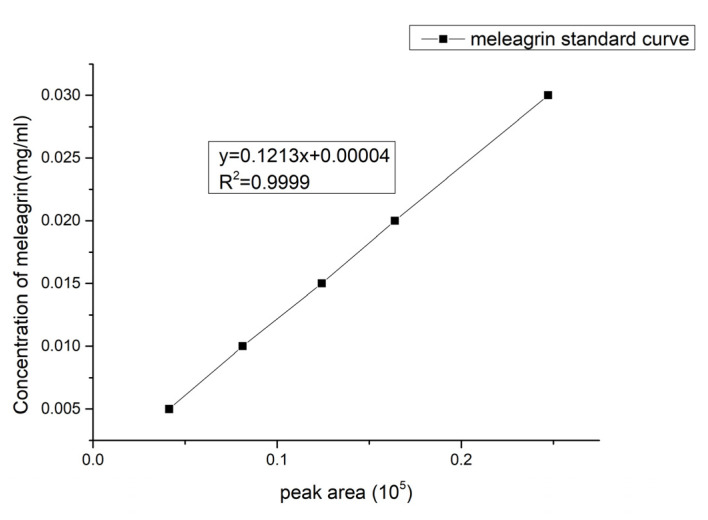
Meleagrin standard curve.

Firstly, the standard curve formula of the compound was established as y = 0.1213x + 0.00004, R^2^ = 0.9999, where x is the peak area and y is the concentration of the compound meleagrin (mg/mL). In Figure 6, the curve showed a good linear relationship and can be used to determine the contents of the compound meleagrin.

### 2.5. Determination of Basic Medium

**Figure 7:**
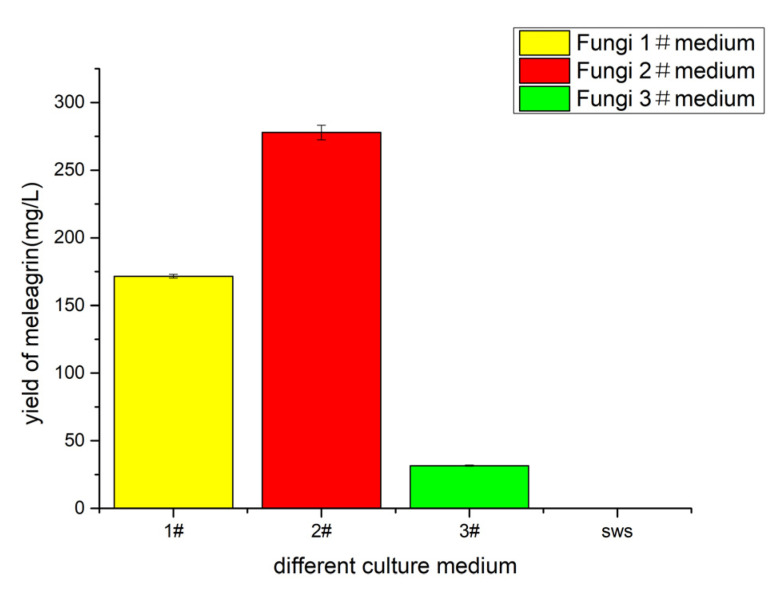
The yield of meleagrin in different fermentation mediums. # represents the number of the culture medium.

Fungi 1# medium, fungi 2# medium, fungi 5# medium, and fungi SWS medium were selected for fermentation culture (Table 3). The initial acidity was 3.0, the static culture was 30 days, the fermentation temperature was 28–30 °C, and the metabolites were extracted with ethyl acetate after fermentation. The crude extract was prepared with methanol at 0.5 mg/mL, the crude extract solution was detected by LC-MS (injection volume of 1 μL), and the yield of meleagrin under different medium conditions was calculated according to the peak area and standard curve of meleagrin. The results (Figure 7) showed that the yield of meleagrin was the highest under the condition of the fungus 2# medium, and the yield of meleagrin was lower under the condition of the fungus 1# and the fungus 5# medium, except that SWS no longer metabolized to produce meleagrin compounds. Therefore, the fungus 2# medium was still used to optimize the yield of compound meleagrin.

### 2.6. Determination of Fermentation Methods

**Figure 8:**
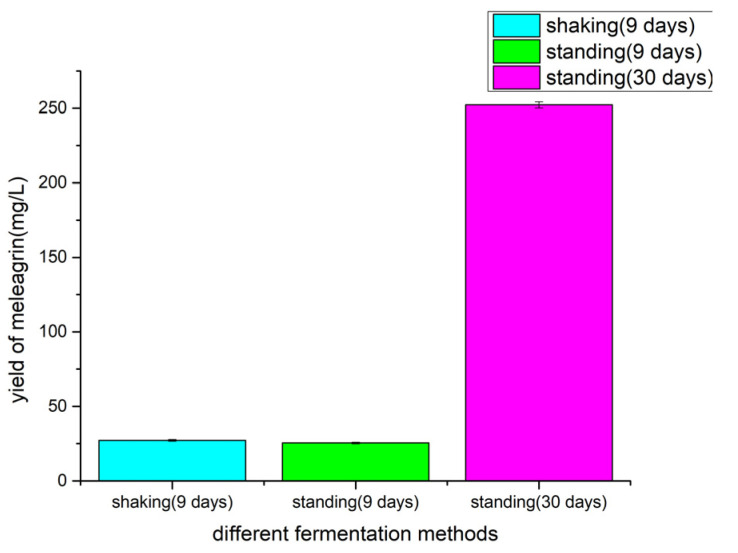
The yield of meleagrin by different fermentation methods.

The Fungi 2# medium, pH 3.0, shaker (180 r/min, 28 °C, fermentation for 9 days) and static (28 °C, fermentation for 9 days, 30 days) were used for fermentation. The crude extract of fermentation was prepared into a 1 mg/mL solution with methanol. The crude extract solution was detected by LC-MS (injection volume 1 μL), and the yield of meleagrin under shaking and standing conditions was calculated according to the standard curve. The effect of culture methods on the yield of meleagrin was investigated, and the results (Figure 8) showed that under the control of other fermentation conditions except for the culture methods, the strain had the highest yield of meleagrin under the static condition and the lowest yield under the shaking culture condition. Therefore, we chose static conditions as the fermentation method for optimization.

### 2.7. Single-Factor Optimization

#### 2.7.1. Effect of Fermentation Days on Meleagrin Yield

The growth or metabolic state of microorganisms is different on different days, which in turn affects the secondary metabolites of microorganisms. Because the biosynthetic pathways of microorganisms are different, it can be considered that the biosynthetic process of microorganisms can be cut off according to preliminary studies, and then the expected metabolites can be obtained [26].

**Figure 9:**
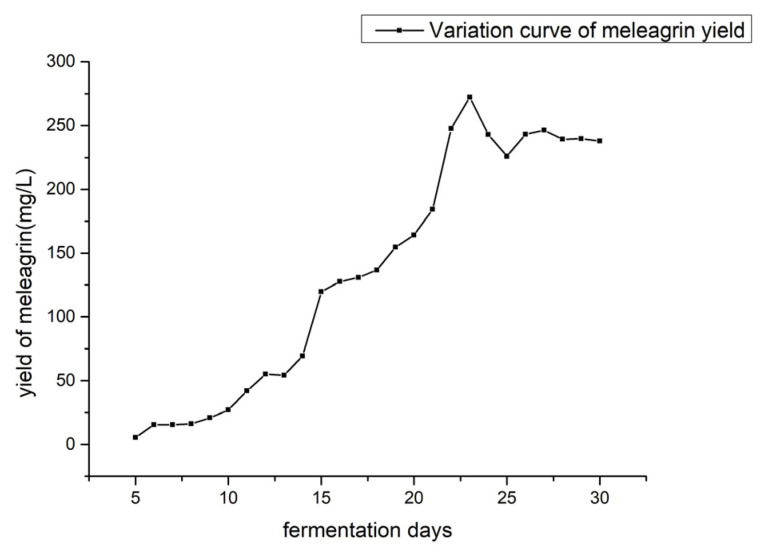
The yield of meleagrain under different fermentation days.

The line chart (Figure 9) showed that the meleagrin production trend of the strain was first increased, then slightly decreased, and then stabilized by analyzing meleagrin production on different days under the fermentation conditions of the fungus 2# medium, acidity 3.0, and standing and culture temperatures of 28–30 °C. At 22–30 days of fermentation, the yield was stable at 225–272 mg/L. From the yield curve, we can choose about 23 days of fermentation as the optimal culture days.

#### 2.7.2. Effect of Initial Fermentation pH on Meleagrin Production

The pH value of microbial growth is very wide, probably between 2–8, but there are only a few microorganisms that can exceed this range, such as acidophilic and basophilic microorganisms, and, for almost all microorganisms, their optimum pH range for growth is between 5–9. In the optimum acidity range, microorganisms grow and reproduce rapidly, and metabolites are abundant. At the same time, an extremely low pH and a high pH also activate or silence the expression of microbial genes in varying degrees, thus affecting the secondary metabolites of microorganisms.

**Figure 10:**
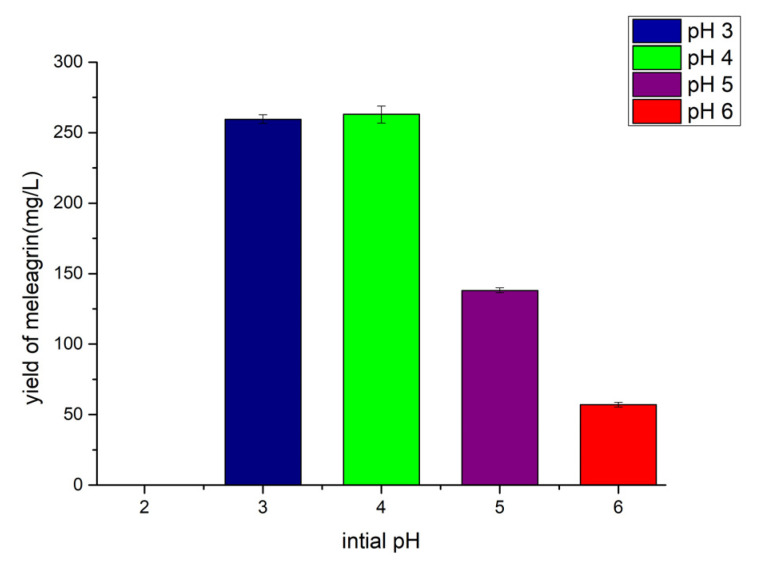
The yield of meleagrin under different pH.

The results (Figure 10) showed that the strain no longer grew under the initial acidity of pH 2.0 and pH 2.5 by controlling other fermentation conditions except for the initial pH of fermentation. The initial acidity of pH 3.0 and pH 4.0 had an effect on the yield of meleagrin. Although the metabolites yield at an acidity of pH 3.0 and pH 4.0 were almost the same, the composition at pH 3.0 was relatively simple, so the yield of meleagrin was optimized at an initial acidity of pH 3.0.

#### 2.7.3. Effects of Different Carbon Sources on Meleagrin Production

According to the above optimization of the medium, the yield of the fungal 2# medium was much higher than that of any other medium, and the design experiment was carried out in combination with its culture components. Among them, the SWS medium did not metabolize to produce meleagrin compounds, and the fungi 5# medium produced less. Here, we compared fungi 1# medium with fungi 2# medium and made a bold guess about the possible reasons that affected the production of meleagrin. Both mediums contained 0.05% KH_2_PO4 and 0.03% MgSO_4_ 7H_2_O, and the content was consistent. On the carbon source, fungus 1# was 2% sorbic alcohol and 2% maltose; the fungi 2# medium contained 2% mannitol, 2% maltose, and 1% glucose. On the nitrogen source, the fungus 1# culture medium contained 1% monosodium glutamate, 0.05% tryptophan, and 0.3% yeast extract; the fungi 2# medium contained 1% monosodium glutamate, 0.1% corn extract, and 0.3% yeast extract.

**Figure 11:**
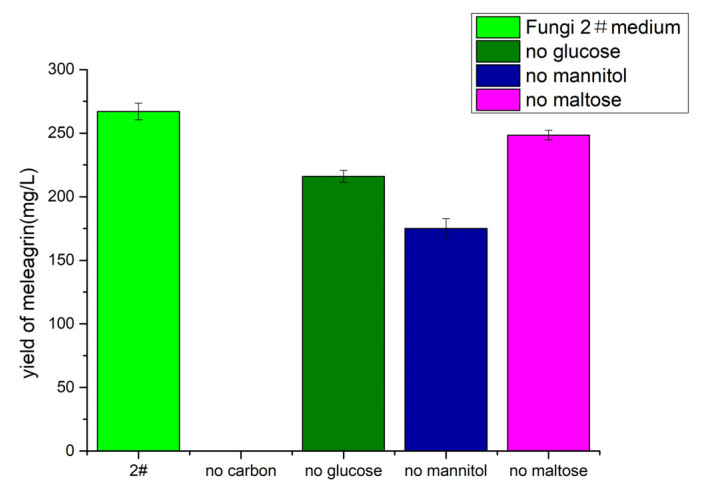
The yield of meleagrin under different carbon sources. # represents the number of the culture medium.

The effect of carbon sources on the yield was investigated. The experimental results (Figure 11) showed that in the absence of glucose, mannitol, and maltose, the strain still produced meleagrin, but in the absence of a carbon source, no meleagrin compounds were produced. This result shows that the carbon source not only provides the necessary nutrients for microbial growth and metabolism, but that the production of compound meleagrin cannot compensate for a lack of a carbon source; different carbon sources have a weak effect on the production of meleagrin.

#### 2.7.4. Effects of Different Nitrogen Sources on Meleagrin Production

**Figure 12:**
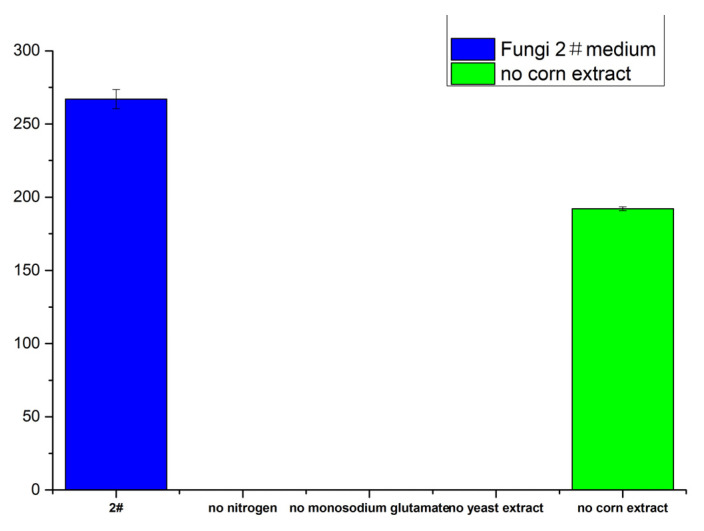
The yield of meleagrin under different nitrogen sources. # represents the number of the culture medium.

The effect of nitrogen sources on the yield was investigated, and the results (Figure 12) showed that meleagrin could still be produced in the absence of corn extract but no meleagrin produced in the absence of monosodium glutamate, yeast extract, or nitrogen sources. The yield of meleagrin in the medium without corn steep liquor was also significantly reduced. This indicates that the nitrogen source was essential for the synthesis of meleagrin compounds.

#### 2.7.5. Effect of Precursor Addition on Meleagrin Production

**Figure 13:**
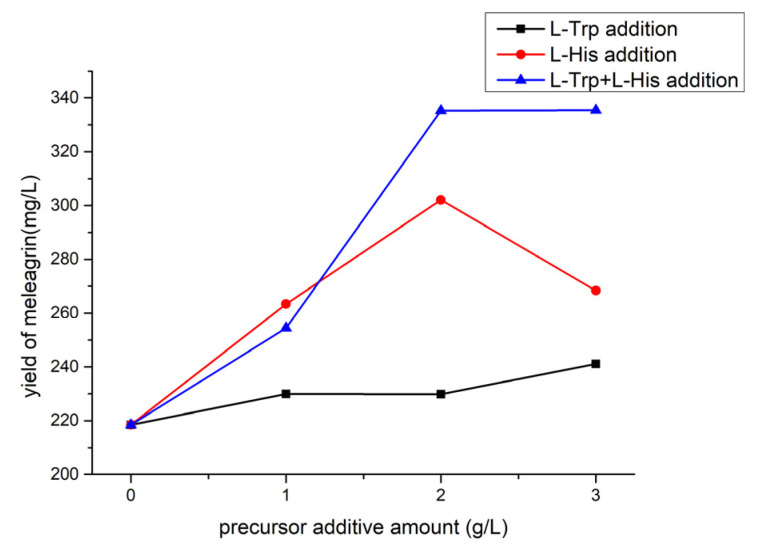
The yield of adding different meleagrin precursors.

Based on the culture of fungal 2#, the effect of adding precursor amino acids to the yield of meleagrin and the utilization of precursors were tested. If the amount of meleagrin increased and L-Try and L-His were utilized, it indicated that L-Try and L-His were involved in the production of secondary metabolites. Different doses of L-Try and L-H were added to the fungi 2# medium to explore whether the precursor addition could be used and what the relationship with the dose was. The experimental results (Figure 13) showed that the addition of precursors could increase the yield of meleagrin compounds to a certain extent. From the perspective of the dose relationship, it is not a linear dose dependence; instead, it first increases and then slows down or even decreases. Overall, the addition of precursors increased the production of meleagrin from 218 mg/L to 335 mg/L.

In conclusion, through the LC-MS analysis of the crude extracts from the previous conditions, the optimal fermentation method was determined to be static with fungal 2# medium and pH 3.0. It is noteworthy that not all the media of the strain can produce meleagrin. We explored the medium that could metabolize the compound in many media. According to the previous optimization conditions, the fermentation number of the strain was further optimized, and the yield of meleagrin was the highest when the fermentation number was 23 days. The yield of meleagrin was increased from 218 mg/L to 335 mg/L by introducing L-histidine and L-tryptophan into the medium.

## 3. Discussion

Hepatocellular carcinoma (HCC) is a kind of malignant tumor. There are about 600,000 new cases of HCC every year in the world, ranking it fifth among malignant tumors [27]. It was reported that many meleagrins and their analogues showed the potential for good anti-tumor activity and antibacterial activity [8,28,29]. The meleagrins and their analogues showed weak cytotoxicity against the A-549 cell line, but they also induced apoptosis in HL-60 cells or arrested the cell cycle through the G2/M phase [3]. The antitumor bioactivities on the human liver carcinoma cell line HepG2 of meleagrin and oxaline have not been reported. In this study, we first reported that meleagrin (**1**) and oxaline (**2**) could effectively inhibit the proliferation and metastasis of human HepG2 cells and induce cell cycle arrest in the G2/M phase and cell apoptosis.

Meleagrin and its derivatives can be used as a class of compounds with great potential in new drug development and ecological management. Takeshi Yamada achieved the total synthesis of Neoxaline, an analog of Meleagrin. The whole process took 60 steps, and the total yield was 7.9%. Though this extremely complex method allowed the preparation of Neoxaline to be greater than 300 mg, this method obviously cannot achieve large-scale production. At the time, the article only described the synthesis process of meleagrin and oxaline and ultimately did not synthesize these two compounds [13]. The chemical synthesis method still has cumbersome steps, a high cost, a low yield, and enantiomers in the final product. Therefore, it is very important to provide a wild-type, high-yield strain that can accumulate a large number of raw materials and meet the research needs. In this study, we found a talent strain with a high yield of compound meleagrin, and the yield of meleagrin increased from 218 mg/L to 335 mg/L by fermentation optimization. Although the fermentation optimization method was used to increase the yield of meleagrin, genetic modifications, metabolic regulation, and a further fermentation optimization method remain to be studied.

## 4. Materials and Methods

### 4.1. Strain

The aciduric fungus *Penicillium* sp. OUCMDZ-1435 was isolated from the mangrove soil (19°34′ N, 110°45′ E) in Wenchang Mangrove Reserve, Hainan Province, China. It was deposited in the China General Microbiological Culture Collection Center (CGMCC), with the preservation number CGMCC No. 40345. It was identified as *Penicillium* sp. by its morphological characteristics and ITS rDNA gene sequences (Figure S1). The strain used was preserved at the Laboratory of the School of Medicine and Pharmacy, Ocean University of China.

### 4.2. Biological Probes

Three pairs of primers were designed as probes to rapidly test whether the existing marine-origin strains in our lab could synthesize roquefortine alkaloids. Three pairs of primers were designed as universal primers based on multiple homologous sequences. (Table 3).

Colonies of pure strains grown on PDA plates were added to 30 μL of DNA lysis solution (Lysis Buffer for Microorganism to Direct PCR, purchased from TaKaRa) using sterile toothpicks, which were vortexed and mixed, reacted in a metal bath at 85 °C for 15 min, and centrifuged at 12000 r/min for 2 min. The supernatant could be used as a PCR reaction template to verify the use of primers by the PCR amplification instrument (BIO-RAD T100 gene amplification instrument). Amplification was detected by agarose gel electrophoresis (Tennant EPS300, Shanghai) and the presence of the target band by a gel imaging analyzer (JS-2000 fully automatic digital gel imaging analyzer), which confirmed the presence of the key enzyme for Meleagrin/Oxaline synthesis in the strain.

### 4.3. Fermentation and Extraction

The OUCMDZ-1435 strain was inoculated on a PDA solid medium with pH 4 for domestication and then inoculated into the fungal 2# liquid medium to obtain the seed liquid. The seed liquid was cultured at 28 °C 180 r/min for 3 days, and the seed liquid was fine and spherical.

Preparation of fungi 2#, pH 4.0, medium, 300 mL/1000 mL, 121 °C, 101 KPa, sterilization 25 min was conducted. The inoculation amount in each bottle was 5%, and the strain was cultured at room temperature on a static shelf. The strain was fermented for 26 days.

After being inactivated with a small amount of ethyl acetate, the fermentation broth layer was separated from the cell layer. The fermentation broth layer was stirred and extracted with ethyl acetate in a large stirring tank three times, and the fermentation broth layer extract was concentrated to 36 g. The mycelium layer was soaked in 80% acetone water solvent, ultrasonically concentrated, dissolved in ethyl acetate, filtered by a Buchner funnel, and concentrated to a 51-g extract.

### 4.4. Isolation and Identification

The comprehensive application of column chromatography (vacuum column chromatography, pressurized column chromatography, normal pressure column chromatography, and different fillers such as silica gel, alumina, and macroporous adsorption resin), gel column chromatography (methanol, dichloromethane-methanol = 1:1, tetrahydrofuran, acetone), and high-performance liquid phase semi-preparative separation methods for compound 1 was isolated with 8.8 mg from the mycelia extract and compound 2 was isolated with 1.5 mg from the fermentation broth extract. The structure of the compounds was determined by comparing the NMR data with the literature.

### 4.5. Cell Culture and Cytotoxicity Assays

A549 (a non-small-cell lung cancer cell line), K562 (a chronic myelogenous leukemia cell line), MCF-7 (an invasive breast ductal carcinoma cell line), P6C (a CD44+ colorectal cancer stem cell line), and HCT-116 (a colon carcinoma cell line) were cultured in an RPMI-1640 medium (Solarbio science & technology, Beijing, China), and HepG2 (Human hepatoma cell line) and L-02 (human embryo liver cell line) were maintained in DMEM medium (Solarbio science & technology, Beijing, China). Cells were incubated in the medium and supplemented with 10% fetal bovine serum (FBS) (Gibco, Carlsbad, CA, USA) and 1% Penicillin-Streptomycin Solution (Solarbio science & technology, Beijing, China). The cells were put in a humidified incubator at 37 °C in 5% CO_2_.

Compounds were evaluated for their cytotoxicity against the A549, P6C, MCF-7, HCT-116, HepG2 and L-02 cell lines, which were evaluated using the MTT (Solarbio science & technology, Beijing, China) colorimetric assay and K562 using the CCK-8 (Beyotime, Shanghai, China) colorimetric assay. The cells (3 × 10^3^/well) were seeded in 96-well plates filled with a culture medium and were then routinely cultured at 37 °C in 5% CO_2_ for 12 h. Then, cells were treated for compounds, and Adriamycin (dissolved in DMSO and diluted with fresh medium) was prepared in various concentrations. After 72 h of treatment, a 20 μL MTT solution (2.5 mg/mL) was added to each well, followed by incubation for 4 h at 37 °C with 5% CO_2_. The MTT solution was removed, and 150 μL DMSO was added to each well to dissolve the MTT that had formed. Absorbance was measured at 570 nm using a microplate reader. Additionally, a 10 μL CCK-8 solution was added to each well after 72 h of treatment on the cells, followed by incubation for 6 h at 37 °C with 5% CO_2_, and absorbance was measured at 450 nm using a microplate reader (Thermo Fisher, Waltham, MA, USA). IC_50_ values were calculated using IBM SPSS Statistics software 24.0(IBM USA).

### 4.6. Proliferation and Metastasis Assay

An MTT (Solarbio science & technology, Beijing, China) assay was used to measure cell proliferation and viability. Briefly, cells were plated in 96-well plates (3000 cells/well) and routinely cultured for 24 h. Then, cells were treated with various concentrations of the compounds meleagrin and oxaline in DMEM supplemented with 10% FBS. After 12 or 24 h of treatment, a 20 μL MTT solution (2.5 mg/mL) was added to each well, followed by incubation for 4 h at 37 °C with 5% CO_2_. The MTT solution was removed, and 150 μL DMSO was added to each well to dissolve the MTT that had formed. Absorbance was measured at 570 nm using a microplate reader.

The logarithmic growth phase of HepG2 cells was plated in 24 plates (10^5^ cells/well), 0.5 ml/well; after the cell fusion degree reached about 80%, it was scratched with a gun head. PBS (Phosphate Buffer Saline Solarbio science & technology, Beijing, China) was then washed again before adding the test compound at a concentration of 1 uM; After 0 h and 48 h of treatment, the plates were observed under a microscope and photographed.

### 4.7. Cell Cycle Analysis

HepG2 cells (10^6^ cells/well) were seeded in six-well plates and placed at 37 °C with 5% CO_2_ in a constant temperature incubator. After 24 h, the supernatant was discarded and treated with meleagrin and oxaline (2.5 to 20 μM) for 24 h. The control group plus 0.5% DMSO. Cells were then harvested, washed, and fixed in 70% ice-cold ethanol at 4 °C for 12 h. Then, the cells were centrifuged, washed with cold PBS, and re-centrifuged. The cells were then re-suspended in a 500 μL cell cycle staining buffer and stained with 25 μL propidium iodide (PI) and 10 μL RNase Dark-proof staining, which took place at room temperature for 30 min. DNA was observed using flow cytometry for cell cycle DNA content analysis to calculate the distribution of the cell cycle in G0/G1, S, and G2/M phases with MultiCycle path.

### 4.8. Cell Death Assay

The cell death assay was performed using an Annexin FITC/PI double staining Apoptosis Detection Kit (Beyotime Biotechnology, Shanghai, China), according to the manufacturer’s instructions. Briefly, cells were cultured on sterile slides in six-well plates over night at 37°C with 5% CO_2_. Then, various dosages of meleagrin and oxaline were added to each well for 24 h. The cells were then washed three times with PBS, harvested 10^6^ cells and re-centrifuged. Then, PBS was discarded and added to the 495 μL binding buffer, 5 μL Annexin V-FITC, and 10 μL PI staining solution in a humidified atmosphere for 15 min at RT in the dark. DNA was observed using flow cytometry to calculate the rate of cells in early apoptosis, necrotic cells, late apoptosis, and viable cells.

### 4.9. Quantitative Detection of Meleagrin and Establishment of the Standard Curve

Purity detection: The compound was identified as meleagrin by mass spectrometry (MS) and nuclear magnetic resonance (^1^H and ^13^C NMR). Then, the compound meleagrin was analyzed by TLC. The developing agents of the two systems (CH_2_Cl_2_-MeOH 10:1, petroleum ether: ethyl acetate = 3:1) and four chromogenic agents (the vanillin-concentrated sulfuric acid universal chromogenic agent, the 254 nm ultraviolet chromogenic agent, the 365 nm fluorescence chromogenic agent, and the bismuth potassium iodide alkaloid chromogenic agent) were used in developing the compound meleagrin. The color showed a single spot; the compound meleagrin was purified by HPLC analysis, and its peak area percentage was greater than 98% at different wavelengths, which met the criteria for establishing a standard curve.

Drawing of the standard curve: The compound meleagrin was detected to meet the standard curve, which was prepared with different concentrations (0.005, 0.010, 0.015, 0.020, and 0.030 mg/mL) of methanol by liquid chromatography analysis and detected by LC-MS using the peak area normalization method. Each sample was injected into 1 μL, and the detection wavelength was 347 nm. The standard curve of meleagrin was made by analyzing the relationship between the peak area and the concentration of meleagrin.

### 4.10. Single-Factor Optimization of Fermentation Conditions

#### 4.10.1. Determination of the Influence of Different Fermentation Days

Fungi 2# medium pH 3.0, static culture, fermentation temperature of 28–30 °C. From the second day, fermentation was carried out every day, and metabolites were extracted with ethyl acetate after fermentation. The crude extract (2–21 days) was prepared into 1 mg/mL with liquid chromatography methanol, and the crude extract (22–30 days) was prepared into 0.1 mg/mL with methanol. The crude extract solution was detected by LC-MS (injection volume 1 μL), and the yield of meleagrin under different medium conditions was calculated according to the peak area and the standard curve of meleagrin.

#### 4.10.2. Determination of the Influence of Different Initial pH

Based on the fungal 2# medium, six concentration gradients of pH 2.0, 2.5, 3.0, 4.0, 5.0, and 6.0 were selected for fermentation cultures. The fermentation temperature was 28–30 °C. After fermentation, the metabolites were extracted with ethyl acetate. The crude extract was prepared with liquid chromatography methanol at 0.5 mg/mL, the crude extract solution was detected by LC-MS (injection volume of 1 μL), and the yield of meleagrin under different medium conditions was calculated according to the standard curve.

#### 4.10.3. Determination of the Influence of Different Carbon Sources

On the basis of the fungal 2# medium, five groups of experiments were set up: 2#, 2# (no carbon source), 2# (no glucose), 2# (no mannitol), and 2# (no maltose). Two parallel experiments in each group included pH 3.0, a temperature of 28–30 °C, and 22–30 culture days. To observe the effect of each carbon source on meleagrin production. The crude extract was prepared at a concentration of 0.5 mg/mL for analysis (injection volume of 1 μL). The yield of meleagrin under different carbon sources was calculated according to the peak area integral and the standard curve of meleagrin.

#### 4.10.4. Determination of the Influence of Different Nitrogen Sources

On the basis of the fungal 2 # medium, five groups of experiments were set up, including 2#, 2# (no nitrogen source), 2# (no monosodium glutamate), 2 # (no yeast extract), and 2# (no corn extract). Two parallel experiments were conducted in each group. A pH of 3.0, temperature of 28–30 °C, and 22–30 culture days showed the effect of each nitrogen source on the yield of meleagrin. The crude extract was prepared at a concentration of 0.5 mg/mL for analysis (injection volume of 1 μL).

#### 4.10.5. Determination of the Influence of Different Precursor Addition Experiments

In the biosynthesis mechanism of meleagrin, L-tryptophan and L-histidine are precursors in the biosynthesis pathway of meleagrin compounds [19]. Therefore, L-tryptophan and L-histidine were added to the medium as precursors to increase the yield of meleagrin compounds. In this way, we designed the following scheme:

Scheme A: L-tryptophan + fungi 2#. Compared with Fungi 2#, the effect of L-tryptophan on the yield was examined.

Scheme B: L-histidine, + fungi 2#. Compared with fungi 2#, the effect of L-histidine on yield was examined.

Scheme C: L-tryptophan, L-histidine, + fungi 2#.

The fungi 2# medium was used as the control.

The crude extract was prepared with methanol at 0.5 mg/mL, and the crude extract solution was detected by LC-MS (injection volume of 1 μL), and the yield of meleagrin under different medium conditions was calculated according to the peak area and the standard curve of meleagrin.

## 5. Conclusions

Roquefortines alkaloids, containing a unique dihydroindole spiroamide framework, showed significant bioactivities. Among them, meleagrin has shown good cytotoxic activity against many tumor cells, revealing its potential in anti-tumor bioactivities. In this article, we describe how our study used PCR probes to search for the fungi bank of our group, *Penicillium* sp. OUCMDZ-1435 was discovered as the target strain, and meleagrin (**1**) and oxaline (**2**) were obtained. Although there are many reports on the biological activities of **1** and **2**, we first reported that meleagrin and oxaline could effectively inhibit the proliferation and metastasis of human HepG2 cells and induce HepG2 cell apoptosis and cell cycle arrest in the G2/M phase. At the same time, meleagrain’s fermentation conditions were optimized, and the yield of meleagrin was increased to 335 mg/L. These results provided bioactive inspiration and a fungus resource for roquefortines alkaloids development, and they also greatly improved their yield and reduced economic costs for scaled application.

## Figures and Tables

**Table 1:** Primers used as biological probes.

Primer	Size (bp)	Sequence (5′-3′)	Product Size (bp)
RDS-F	21	AGCTATGGCTTTGATAGCAGC	567
RDS-R	21	CACCAGATCGCCGGTTTTATA	
RPT-F	20	AGCGTGGAACTGAGCCAGAA	567
RPT-R	21	CCAATGTTCTTCCACTTTCGC	
SRO-F	20	TGACCACGCGAGCATTTGTC	501
SRO-R	20	TCATCACGCTCTCCATGAGT	

**Table 2:** Cytotoxicities of meleagrin and oxaline (IC_50_, μM).

Cell line	A549	MCF-7	P6C	K562	L-02
meleagrin	>10	4.94 ± 0.001	>10	>10	>10
oxaline	6.85 ± 0.03	>10	9.41 ± 0.01	7.8 ± 0.02	>10
Adriamycin	0.26 ± 0.006	0.82 ± 0.014	0.4 ± 0.001	0.92 ± 0.04	0.21 ± 0.01
Cell line	HCT-116	HePG2			
meleagrin	5.7 ± 0.013	1.82 ± 0.021			
oxaline	4.94 ± 0.001	4.27 ± 0.01			
Adriamycin	0.21 ± 0.01	0.04 ± 0.002			

**Table 3:** Culture medium constituents.

Medium	Medium Constituents
SWS	peptone 0.1%, soluble starch 1%, seawater
1#	sorbic alcohol 2%, maltose 2%, monosodium glutamate 1%, KH_2_PO_4_ 0.05%, MgSO_4_ 7H_2_O 0.03%, tryptophan 0.05%, yeast extract 0.3%, seawater
2#	monosodium glutamate 1%, mannitol 2%, maltose 2%, KH_2_PO_4_ 0.05%, glucose 1%, corn extract 0.1%, MgSO_4_ 7H_2_O 0.03%, yeast extract 0.3%, seawater
5#	glucose 2.0%, peptone 1%, malt leaching powder 0.3%, yeast extract 0.3%, seawater

# represents the number of the culture medium.

## Data Availability

Not applicable.

## Supplementary Materials

The following supporting information can be downloaded at: https://www.mdpi.com/article/10.3390/molecules28073180/s1. Figure S1: Phylogenetic relationship based on ITS rDNA gene sequences using the neighbor-joining method; Figure S2: The biosynthetic pathway of diketopiperazine alkaloids; Figure S3: ^1^H(400MHz)-NMR spectrum of compound 1 in DMSO; Figure S4: ^13^C(125MHz)-NMR spectrum of compound 1 in DMSO; Figure S5: ^1^H(500MHz)-NMR spectrum of compound 2 in DMSO; Figure S6: ^13^C(125MHz)-NMR spectrum of compound 2 in DMSO; Table S1: ^1^H and ^13^C NMR Data for Compounds 1 and 2 (^1^H 500MHz, ^13^C 150 MHz, DMSO-d6, TMS, δ ppm).

## References

[1] Kozlovskii A.G., Zhelifonova V.P., Antipova T.V. (2013). Fungi of the genus Penicillium as producers of physiologically active compounds (Review). Appl. Biochem. Microbiol..

[2] Zeilinger S., Martín J.F., García-Estrada C., Zeilinger S. (2014). Biosynthesis and Molecular Genetics of Fungal Secondary Metabolites. Fungal Biology.

[3] Du L., Feng T., Zhao B. (2010). Alkaloids from a deep ocean sediment-derived fungus *Penicillium* sp. and their antitumor activities. J. Antibiot..

[4] Clark B., Capon R. (2005). Roquefortine E, a diketopiperazine from an Australian isolate of *Gymnoascus reessii*. J. Nat. Prod..

[5] Ohmomo S., Sato T., Utagawa T. (1975). Isolation of festuclavine and three new indole alkaloids, roquefortine A, B and C from cultures of Penicillium roqueforti. Agric. Biol. Chem..

[6] Ohmomo S., Oguma K., Ohashi T. (1978). Isolation of a new indole alkaloid, roquefortine D, from the cultures of *Penicillium roqueforti*. Agric. Biol. Chem..

[7] Ries M.I., Ali H., Lankhorst P.P. (2013). Novel key metabolites reveal further branching of the roquefortine/meleagrin biosynthetic pathway. J. Bio. Chem..

[8] Niu S., Wang N., Xie C.L. (2018). Roquefortine J, a novel roquefortine alkaloid, from the deep-sea-derived fungus *Penicillium granulatum* MCCC 3A00475. J. Antibiot..

[9] Garcia-Estrada C., Ullan R.V., Albillos S.M., Fernandez-Bodega M.A. (2011). A single cluster of coregulated genes encodes the biosynthesis of the mycotoxins roquefortine C and meleagrin in *Penicillium chrysogenum*. Chem. Biol..

[10] Yan Q., Carroll P.J., Winkler J.D. (2019). A Transannular Rearrangement Reaction of a Pyrroloindoline Diketopiperazine. Org. Lett..

[11] Steyn P.S. (1970). The isolation, structure and absolute configuration of secalonic acid D, the toxic metabolite of *Penicillium oxalicum*. Tetrahedron.

[12] Hirano A., Iwai Y., Masuma R. (1979). Neoxaline, a new alkaloid produced by *Aspergillus japonicus*. Production, isolation and properties. J. Antibiot..

[13] Takeshi Y., Ideguchi-Matsushita T. (2015). Asymmetric Total Synthesis of Indole Alkaloids Containing an Indoline Spiroaminal Framework. J. Chem. Eur..

[14] Koizumi Y., Arai M., Tomoda H. (2004). Oxaline, a fungal alkaloid, arrests the cell cycle in M phase by inhibition of tubulin polymerization. Biochim. Biophys. Acta (BBA) Mol. Cell Res..

[15] Reshetilova T.A., Vinokurova N.G., Khmelenina V.N., Kozlovsky A.G. (1995). The role of roquefortine in the synthesis of alkaloids meleagrin, glandicolines A and B, and oxaline in fungi *Penicillium glandicola* and *P. atramentosum*. Microbio.

[16] Gober M., Carroll J., Joullié M. (2016). Triazaspirocycles: Occurrence, Synthesis, and Applications. Mini-Rev. Org. Chem..

[17] Newmister S.A., Stelamar R., Jennifer J.S. (2018). Unveiling sequential late-stage methyltransferase reactions in the meleagrin/oxaline biosynthetic pathway. Org. Biomol. Chem..

[18] Hazrat A., Ries M.I., Nijland J.G. (2013). A Branched Biosynthetic Pathway Is Involved in Production of Roquefortine and Related Compounds in Penicillium chrysogenum. PLoS ONE.

[19] Kosalková K., Domínguez-Santos R. (2015). A natural short pathway synthesizes roquefortine C but not meleagrin in three different *Penicillium roqueforti* strains. Appl. Microbiol. Biot..

[20] De Faveri R., Nunes R., Santin J.R. (2018). The role of kinins in the proliferation of fibroblast primed with TNF in scratch wound assay: Kinins and cell proliferation. Int. Immunopharmacol..

[21] Li Y., Chen D., Su Z. (2016). MicroRNA-106b functions as an oncogene in renal cell carcinoma by affecting cell proliferation, migration and apoptosis. Mol. Med. Rep..

[22] Luo H., Li J., Lin Q. (2020). Ultrasonic irradiation and SonoVue microbubbles-mediated RNA interference targeting PRR11 inhibits breast cancer cells proliferation and metastasis, but promotes apoptosis. Biosci. Rep..

[23] Tang J.J., Zhang Y. (2016). Natural Products as Sources of New Fungicides (III): Antifungal Activity of 2,4-Dihydroxy-5-Methylacetophenone Derivatives. Bioorg. Med. Chem. Lett..

[24] Kim J.A., Cárcer G.D. (2013). Correction to Low Dose of Amino-Modified Nanoparticles Induces Cell Cycle Arrest. Nano.

[25] Yin H., Zhang M.J., An R.F., Zhou J. (2020). Diosgenin Derivatives as Potential Antitumor Agents: Synthesis, Cytotoxicity, and Mechanism of Action. J. Nat. Prod..

[26] Katsuyama Y. (2019). Mining novel biosynthetic machineries of secondary metabolites from actinobacteria. Biosci. Biotech. Bioch..

[27] Gaytan-Graham S. (2001). Hepatocellular carcinoma: An update. Ultrastruct. Pathol..

[28] Mady M.S., Mohyeldin M.M., Ebrahim H.Y. (2016). The indole alkaloid meleagrin, from the olive tree endophytic fungus *Penicillium chrysogenum*, as a novel lead for the control of c-Met-dependent breast cancer proliferation, migration and invasion. Bioorg. Med. Chem..

[29] Du L., Li D.H., Zhu T.J., Wang F.P. (2009). GuNew alkaloids and diterpenes from a deep ocean sediment derived fungus *Penicillium* sp. Tetrahedron.

