# Supplementary material for: Discovery, Antitumor Activity, and Fermentation Optimization of Roquefortines from Penicillium sp. OUCMDZ-1435
Source: Molecules. 2023 Apr 3;28(7):3180. doi: 10.3390/molecules28073180 (PMC10095773; doi:10.3390/molecules28073180)
Supplement: Supplementary file 1 [file molecules-28-03180-s001.zip › molecules-2297310-supplementary.pdf]

Supporting Information

Discovery, Antitumor Activity and Fermentation Optimization of Roquefortines from Marine-derived Fungi

Xingxing He [1†], Yanzheng Jin [1†], Fei Kong [1†], Liyuan Yang [1], Mingzhuang Zhu [2] and Yi Wang [1,*]

List of Supporting Information

Figure S1. Phylogenetic relationship based on ITS rDNA gene sequences using the neighbor-joining method. **S2**

Figure S2. The biosynthetic pathway of diketopiperazine alkaloids **S2**

Figure S3. ^{1}H(400MHz)-NMR spectrum of compound 1 in DMSO **S3**

Figure S4. ^{13}C(125MHz)-NMR spectrum of compound 1 in DMSO **S3**

Figure S5. ^{1}H(500MHz)-NMR spectrum of compound 2 in DMSO..**S4**

Figure S6. ^{13}C(125MHz)-NMR spectrum of compound 2 in DMSO **S4**

Table S1. ^{1}H and ^{13}C NMR Data for Compounds 1 and 2（^{1}H 500MHz, ^{13}C 150 MHz, DMSO-d6, TMS, δ ppm） **S5**

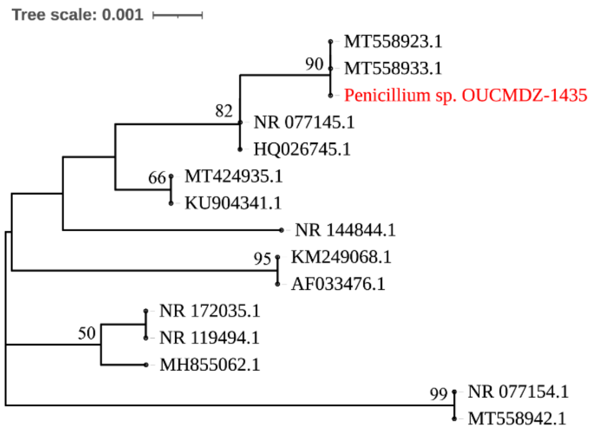

Figure S1.: Phylogenetic relationship based on ITS rDNA gene sequences using the neighbor-joining method. The scale bar represents 0.001 substitutions per nucleotide. Strain Number Notes: Penicillium goetzii isolate 2010F20 (MT558933.1),Penicillium rubens isolate 2010F5 (MT558923.1), Penicillium chrysogenum CBS 306.48 (NR_077145.1), Penicillium chrysogenum strain ATCC 10106 (HQ026745.1), Penicillium fimorum strain DTO149-B8 (MT424935.1), Penicillium fimorum strain CBS 140575 (KU904341.1), Penicillium compactum CGMCC 3.15411 (NR_144844.1), Penicillium cellarum isolate F727 (KM249068.1), Penicillium aurantiogriseum strain NRRL 971 (AF033476.1), Penicillium speluncae DAOMC 251701 (NR_172035.1), Penicillium solitum FRR 937 (NR_119494.1), Penicillium fuscoglaucum culture CBS:261.29 (MH855062.1), Penicillium expansum ATCC 7861 (NR_077154.1), Penicillium fimorum isolate 2011F12 (MT558942.1).

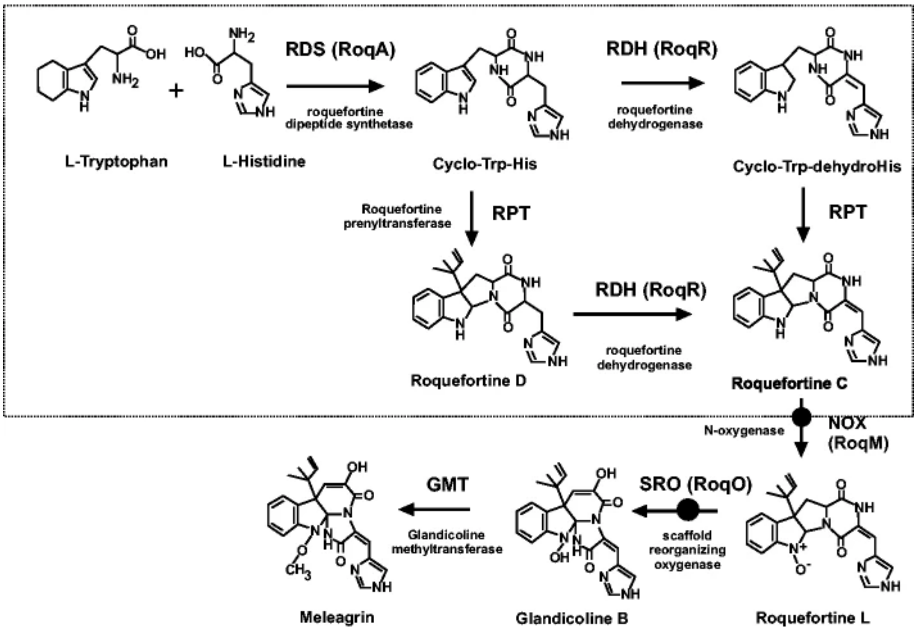

Figure S2. The biosynthetic pathway of diketopiperazine alkaloids[1]

[1]Kosalková, K.; Domínguez-Santos, R. A natural short pathway synthesizes roquefortine C but not meleagrin in three different Penicillium roqueforti strains. Appl Microbiol Biot2015,99,7601-7612.

Figure S3. ^{1}H(400MHz)-NMR spectrum of compound **1** in DMSO

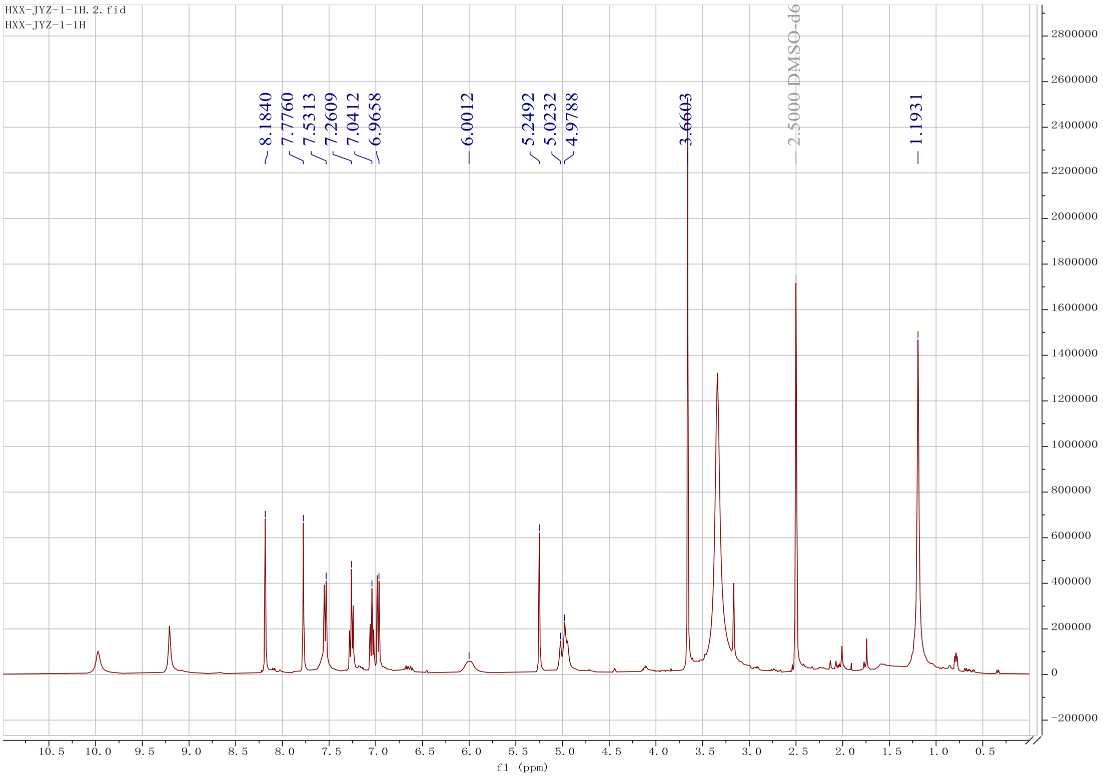

Figure S4. ^{13}C(125MHz)-NMR spectrum of compound **1** in DMSO

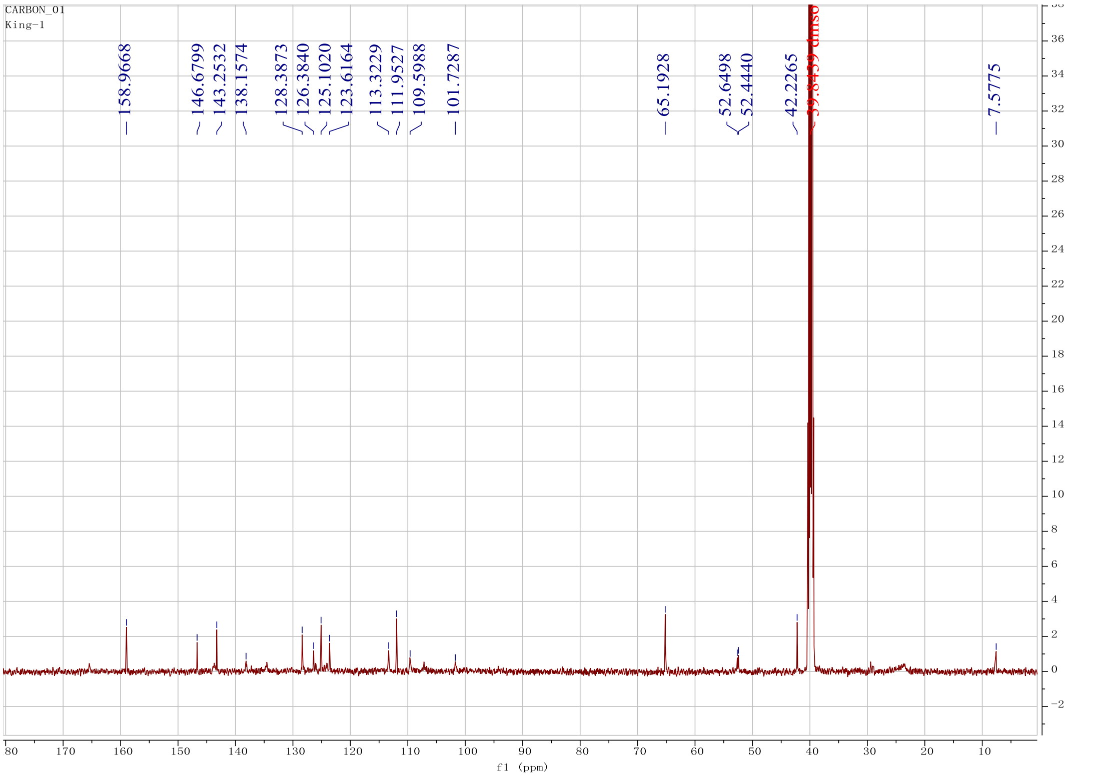

Figure S5. ^{1}H(500MHz)-NMR spectrum of compound **2** in DMSO

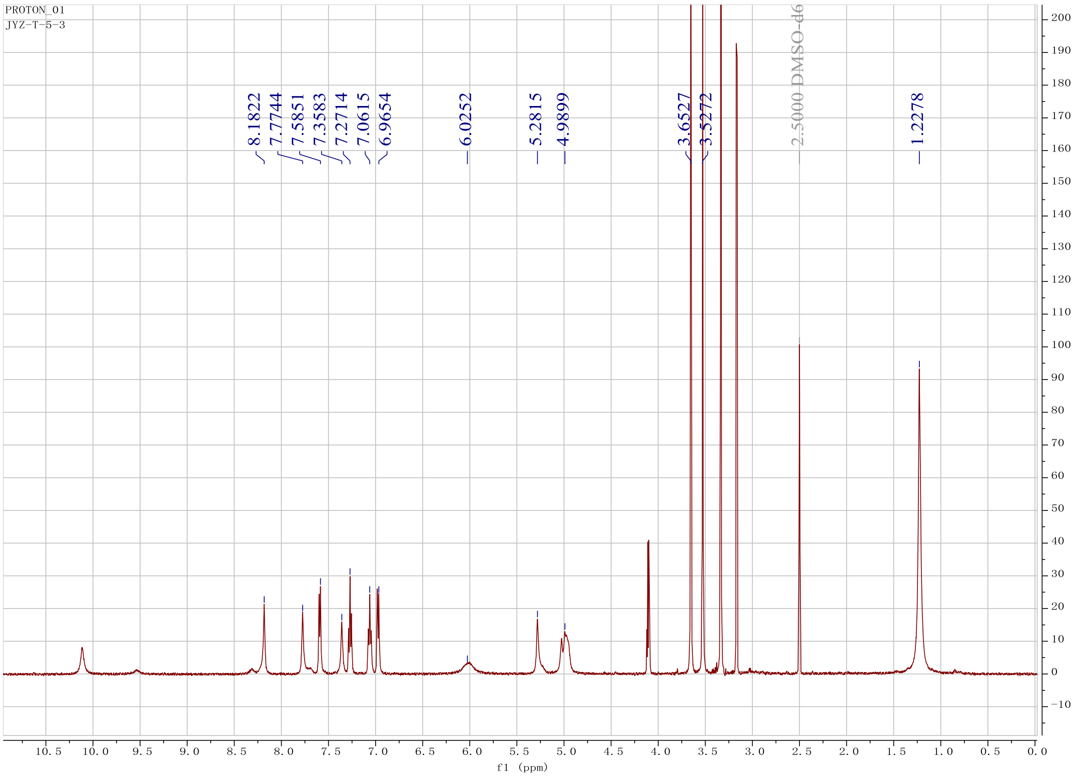

Figure S6. ^{13}C(125MHz)-NMR spectrum of compound **2** in DMSO

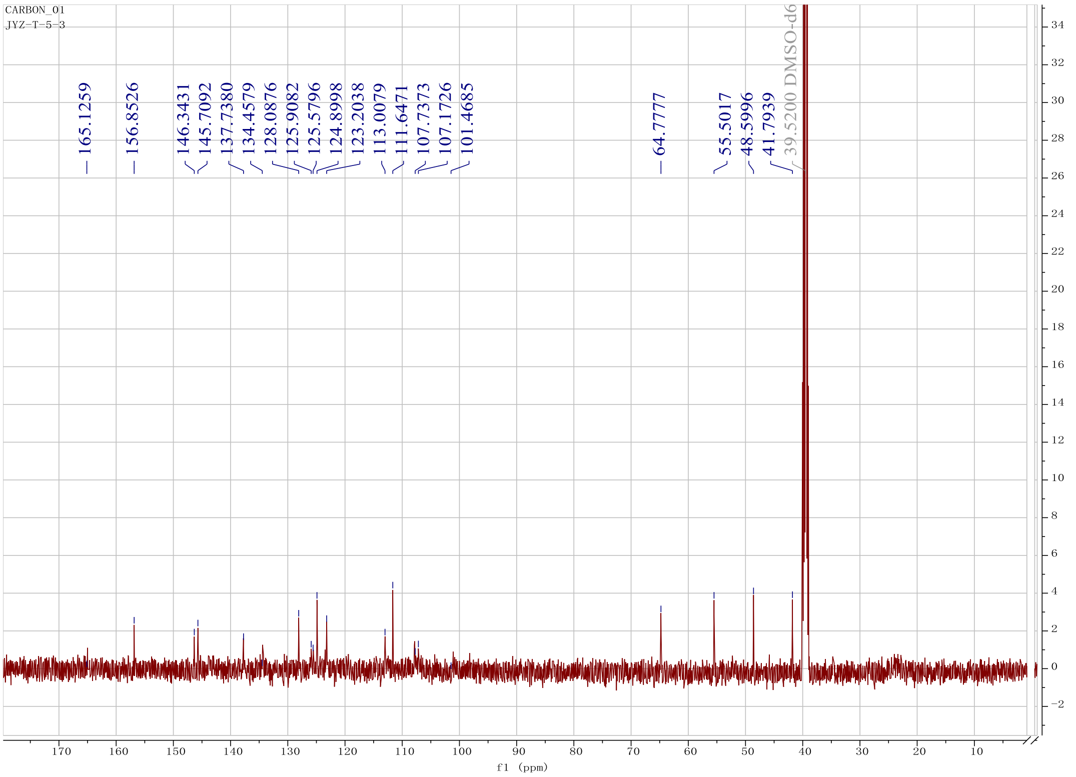

Table S1. ^{1}H and ^{13}C NMR Data for Compounds **1** and **2**（^{1}H 500MHz, ^{13}C 150 MHz, DMSO-d6, TMS, δ ppm）

Position	Compound 1		Compound 2	
	δ_C	δ_H (*J* in Hz)	δ_C	δ_H (*J* in Hz)
1		3.66， s		3.65， s
2	101.7,C		101.1,C	
3	52.5,C		55.5,C	
4	125.1,CH	7.53,d	124.9,CH	7.59,d
4a	126.4,C		125.5,C	
5	123.7,CH	7.04,t	123.2,CH	7.06,t
6	128.4,CH	7.26,t	128.1,CH	7.27,t
7	111.9,CH	6.97, d	111.6,CH	6.97, d
7a	146.6,C		146.3,C	
8	109.3,CH	5.25,s	107.7,CH	5.28,s
9	143.2,CH		142.9,C	
9’			64.7,CH_3	3.53,s
10	158.9,C		158.8,C	
11				
12	123.9,C	3.82, s	124.8,C	
13	165.4,C		165.0,C	
14				10.11,s
15	107.2,CH	8.18,s	107.1,CH	8.18,s
16	126.3,C		125.9,C	
17				
18	134.5,CH	7.34,s	134.4,CH	7.36,s
19				
20	138.5,CH	7.78,s	137.7,CH	7.78,s
21	41.9,C		41.8,C	
22	143.7,CH	6.00	143.8,CH	6.01,s
23	113.3,CH	4.98<br>5.02	113.0,CH	5.00<br>5.28
24	23.7,CH_3	1.19,s	24.0,CH_3	1.23,s
25	23.7,CH_3	1.19,s	24.0,CH_3	1.23,s
